# Penalty or premium for foreign ownership in Thailand? Comparing public support for waste-to-energy plants operated by Thai, Japanese, and Chinese Companies

**DOI:** 10.1371/journal.pone.0328165

**Published:** 2025-07-10

**Authors:** Azusa Uji, Jaehyun Song, Nives Dolšak, Aseem Prakash

**Affiliations:** 1 Graduate School of Law, Kyoto University, Kyoto, Japan; 2 Faculty of Informatics, Kansai University, Osaka, Japan; 3 School of Marine and Environmental Affairs, University of Washington, Seattle, Washington, United States of America; 4 Department of Political Science, University of Washington, Seattle, Washington, United States of America; Universidad Nacional de Chimborazo, ECUADOR

## Abstract

Waste-to-energy (WtE) projects use municipal waste to generate energy. While they increase local environmental and health risks, they also generate local environmental and economic benefits. If facility operator characteristics influence perceptions of risks and benefits, does public support for a WtE facility change if it is operated by a foreign firm? Using a survey experiment in Thailand (n = 829), we examine support for a hypothetical WtE plant operated by local Thai (reference category), Japanese (treatment 1), and Chinese (treatment 2) firms. We find that while respondents are less supportive of WtE plants operated by Chinese companies (“penalty for foreignness”), their support does not change for Japanese plants (neither penalty nor premium for foreignness). Importantly, while perceptions of local *economic* benefits increase support, perceptions of *environmental* benefits from reducing waste and landfills do not. Respondents support WtE plants when their communities experience air pollution from coal-fired power plants and when they support the Thai government’s decision to ban the manufacturing and sale of single-use plastics.

## Introduction

Waste-to-energy (WtE) facilities use municipal waste to generate energy. While they increase local environmental and health risks, they also generate local environmental and health benefits. Facility operators tend to claim that their facilities employ state-of-the-art pollution control technologies and generate new jobs. However, it is unclear whether local communities believe their claims about safe operations, pollution control, and economic contributions. Specifically, does the public have more (or less) trust in local firms versus foreign firms? In this paper, we examine whether public support for a hypothetical WtE facility depends on the nationality of the facility operator.

Our paper engages with the broader issue of information problems in the policy process. Scholars note that public opinion can influence public policy, especially in democracies. Indeed, this is why there is a flourishing opinion poll industry, and major media outlets regularly cover the findings of recent public opinion polls. While the public certainly has opinions and policy preferences, it is less clear the extent to which respondents are sufficiently well informed about policy issues. They make cognitive shortcuts, employing stereotypes and heuristics. In our context, boundedly rational individuals [1] probably do not have sufficient knowledge about the health and safety records of specific firms that operate WtE facilities [2,3], especially foreign firms.

In response to this information problems, respondents could make at least two cognitive shortcuts. First, they could assume that all “foreign companies” will use dirty technologies, leading to local pollution and health problems. This speaks to the argument that to attract foreign capital, governments will weaken environmental regulations or not enforce them [4,5]. Seeking lower regulatory costs, profit-seeking companies will establish facilities in “pollution havens” abroad [6–10]. This dynamic is evident in the context of global waste trade. Global North countries ship their plastic waste abroad because of stringent domestic laws as well as the saturation of landfill capacities [11,12].

In many instances, Global South countries do not have the local capacity to appropriately process this waste, thereby causing local health and environmental problems. WtE technology emerges as a win-win solution because it solves the local landfill problem and, at the same time, generates energy. The policy problem is that communities may not have the tools to assess WtE facilities’ local pollution impact. Specifically, they may not have the tools and resources to assess whether multinational corporations operating WtE facilities are using state-of-the-art pollution control technologies and complying with local laws. Indeed, they might suspect that multinational corporations’ WtEs will cause local pollution because these companies are not locally rooted, and local officials, who are eager to attract foreign investments, have weak incentives to enforce anti-pollution laws. This speaks to the literature on multinationals’ overseas operations facing a “liability of foreignness” [13–15], which imposes a higher threshold on them to secure the social license to operate [16,17].

Individuals could take a more nuanced view of foreign companies as well. From this perspective, they might expect that companies coming from “clean countries” will probably use cleaner technologies and show a higher commitment to local pollution laws. The reason is that home country regulations shape corporate practices and culture, which companies seek to replicate in their overseas facilities [18–20]. If so, for local communities, the “country of origin” [21–23] of the facility operator could influence their support for the WtE facility because it serves as a proxy for the facilities’ local environment and health impacts.

Our paper also speaks to the literature on the not-in-my-backyard (NIMBY) phenomenon [24,25]. Some projects create public goods but impose environmental and health risks on local communities (sometimes underprivileged communities, as the environmental justice literature has documented [26]), thereby motivating public mobilization against these projects. This opposition is visible in the context of municipal waste management and the location of hazardous waste facilities [27–29]. Of course, affluent communities also weaponize NIMBYism by employing zoning laws to oppose infill development or public infrastructure projects [30,31]. However, there is less work on linking facility operator characteristics with NIMBYism. Our contribution is to assess local opposition in the context of operator characteristics (holding project characteristics constant), specifically nationality. In doing so, we contribute to a new way of thinking about NIMBYism where firm characteristics could provoke local opposition to the proposed project.

Scholars also note instances of yes-in-my-backyard (YIMBY) where local communities support “undesirable” industries to secure local economic benefits such as employment. While WtE projects increase local pollution risks, they might reduce other environmental risks (such as emissions from coal plants that WtE might replace) or landfill pollution. Thus, in shaping public support, both NIMBY/YIMBY dynamics might operate together, motivated by concerns about both local risks and benefits.

Empirically, we examine public support for a hypothetical WtE plant in Thailand. In 2021, Thailand generated approximately 25 million tons of municipal solid waste [32], but only 16% of the municipal waste was recycled, and 37% was properly disposed of (through landfills, WtE incineration, or composting). Plastic waste accounts for 28% of municipal waste, most of which is deposited in landfills. This is problematic as it takes hundreds of years for plastics to break down [33]. And when they do break down into tiny toxic particles, it causes contamination of the soil and waterways and may even enter the food chain.

About 79 WtE plants already operate in Thailand. But to manage the growing volume of plastic waste, the Thai government seeks to construct additional plants and has allocated US$106.5 million to fund them [34]. However, this policy has faced public opposition due to concerns about local health and environmental risks [35]. For example, in 2022, a member of the provincial council for Mueang District and over 100 residents from Ban Krang subdistrict submitted a petition to the Governor of Phitsanulok province opposing the construction of a proposed WtE plant [36]. In August 2023, more than 100 villagers from Non Thai and Kham Sakae Saeng districts in Nakhon Ratchasima Province staged a public protest against a proposed WtE plant [37]. There are media reports of similar protests in other provinces such as Krabi, Phetchaburi, and Songkhla.

It is not clear if local opposition to WtE depends, in part, on who operates the facility. In an online survey experiment on a representative sample of Thai respondents (n = 829), we assessed public support for a hypothetical WtE plant operated by local (reference category), Japanese, or Chinese firms. We focused on China and Japan as both seek to expand their influence in Asia, often through economic ties. China launched its Belt and Road Initiative (BRI) in 2013 and established the Asian Infrastructure Investment Bank in 2014 to fund infrastructure projects [38–40]. In response, in 2015 Japan initiated the Partnership for Quality Infrastructure, the largest foreign aid expansion program to support infrastructure projects in Asia [40].

Japan and China also compete in green technologies, which are expected to facilitate a transition to a low-carbon economy [41]. Given Chinese companies’ increasing competence in renewable energy, some view BRI as a “green” initiative [42]. Nonetheless, Chinese infrastructure is sometimes characterized as low quality and employing polluting technologies [43–46]. Chinese firms also sometimes do not invest in maintaining good relations with the local community. In contrast, since the 1980s, Japan has been considered a manufacturing powerhouse across sectors ranging from electronics to automobiles [47]. Its products are reliable, and Japanese technology has a reputation for maintaining high quality and low environmental impact. Japanese firms are also viewed as respectful of local laws and communities.

Our online survey experiment suggests that the overall support for WtE plants is relatively high: the mean value is 5.3 on a 1–7 scale. The public is less supportive of WtE plants when they are operated by Chinese companies (as opposed to local companies). Japanese plants enjoyed neither the premium nor faced the penalty of foreign ownership. Thus, the liability of foreignness manifests for Chinese WtE plants only.

Might economic or environmental benefits drive local YIMBY-type support? We found that support is higher among respondents who view WtE plants to be creating local economic benefits. However, support did not change among those viewing WtE plants to generate local environmental benefits from reducing pressure on landfills. However, respondents support WtE plants when their communities experience air pollution from coal-fired power plants and when they support the Thai government’s recent ban on single-use plastics. Thus, the role of local environmental benefits in driving public support is mixed: there is a lack of support for reducing landfill pollution, but an uptick in support for reducing air pollution from coal plants.

## Theory and hypotheses

Municipal waste has emerged as a serious issue in the Global South countries with growing populations, rapid urbanization, poor enforcement of environmental laws, coupled with permissive plastic waste import policies. Historically, these countries have used landfills for waste disposal. However, as the space available for landfills is diminishing, many countries are considering WtE technologies [48–51]. Given the high volume of municipal waste in the Global South, many multinational companies, such as Japan’s Hitachi Zosen, British Allied Project Services, and French ENGIE and Suez Environment, are exploring opportunities to establish WtE plants [52,53]. However, given the local health and environmental risk that such facilities might pose, some communities oppose them. Thus, by focusing on the nationality of the plant operator as a driver of risk-reward perceptions, our paper connects a local NIMBY/YIMBY phenomenon to international politics.

Our focus on the nationality of the WtE operator provides the opportunity to explore how local communities are responding to se to the rise of China as a major exporter of technology and funder of infrastructure development. Under the auspices of the Belt and Road Initiative (BRI), China has funded projects to exploit local resources and tie their financing to the import of Chinese products and services [54]. Some local communities view this as neocolonialism as opposed to ‘win–win’. Indeed, scholars note the rise of anti-Chinese populism in Africa [55–57] and Latin America [58].

In particular, communities worry about the negative impact of Chinese projects on the environment and labor standards [38,59]. In Chinese infrastructure projects, project developers tend to push for quick sign-offs on project contracts. Unlike Western financial institutions, Chinese banks do not impose strict environmental and social conditionalities [60]. Thus, there is an emerging perception that BRI projects are not subject to proper impact assessment [61], at least not as per World Bank standards. Taken together, some local communities view Chinese infrastructure projects as eroding regulatory protections, diminishing fiscal discipline, and government accountability [62–64]. In Thailand, even though policy makers are relatively supportive of BRI, local communities in the Eastern Economic Corridor (EEC) are concerned about environmental pollution from Chinese factories.

In contrast, local communities perceive Japanese infrastructure projects as being more attentive to local laws and using state-of-the-art technology. This is probably why Japan has strategically emphasized the *quality of* infrastructure to differentiate itself from China’s emphasis on the *quantity* of infrastructure [60]. Moreover, under the Partnership for Quality Infrastructure, Japan has emphasized guidelines for environmental and social impact as one of the five components of quality infrastructure investment [60,65].

### Key hypothesis: Liability of foreignness

The above discussion suggests that the “liability of foreignness” might depend on the nationality of the multinational firm operating the WtE facility (with a local Thai operator as a reference category). Thus, we propose:

H1a: Public support is higher for WtE facilities operated by Japanese companies.
H1b: Public support is lower for WtE facilities operated by Chinese companies.

### Additional hypotheses: Not-in-my-backyard

Waste processing projects provoke NIMBY because they provide public goods of reducing waste burden and energy production whose benefits spill over beyond the local jurisdictions, while increasing environmental and health risks for the local communities where the plants are located [66–69]. Scholars have documented NIMBY opposition in the context of nuclear waste repositories [70,71], solid waste/disposal facilities [72], and hazardous waste facilities [73]. But NIMBY extends beyond waste disposal. Scholars note NIMBY opposition to other types of projects including wind energy [25,74–77], solar farms [78], and nuclear energy [78–84].

WtEs could create local environmental and health risks motivating NIMBY opposition. Burning waste releases dangerous chemicals such as dioxins, arsenic, beryllium, cadmium, chromium, lead, mercury, fine particulate matter, and acidic gases. In particular, dioxins can cause cancer and abnormalities in living organisms [85,86]. Given these potential negative local impacts some scholars have also examined the NIMBY opposition in the context of WtE plants [87,88] in China (e.g., [89,90]), Portugal [88], Greece [91], Ireland [92], Italy [93], and Canada [3]. Echoing the findings of the broader NIMBY literature, these scholars note perceptions about risk, trust, economic benefit, and fairness drive this opposition [94]. Some individuals might oppose WtE because they have experienced or heard about the ill effects of any type of waste incineration facility [12,95,96].

H2a: Public support for WtE facilities is lower when respondents believe that their community is exposed to air pollution from existing waste incineration facilities.

Arguably, WtE could replace coal-fired plants. Given the long history of coal, many communities recognize its impact on air pollution [97–99]. While coal-based power plants face pushback due to climate change reasons in the Global North, in the Global South, public resistance is often motivated by their health and environmental impacts. This has led to the cancellation or suspension of plants across Asia, including the Philippines, Thailand, and Vietnam [100]. Thus, those perceiving a negative impact of coal might view WtE as a lower-risk option. Accordingly, we expect:

H2b: Public support for WtE facilities is higher when respondents perceive that their community is exposed to air pollution from coal-fired power plants.

### Additional hypotheses: Yes-in-my-backyard

Notwithstanding the risk perception, local communities sometimes support “undesirable” or “dirty” industries in their backyards, primarily to secure local economic benefits [70,101–107]. WtE plants could motivate YIMBYism because local communities might welcome the local benefits they create, as evident in some cases of nuclear plants [107], prisons [108], and oil pipelines [103]. Some Japanese WtE project developers highlight that waste incineration facilities are often constructed together with waste collection and sorting services, all of which generate employment. Thus, we expect that new jobs and investment opportunities can elicit local support for the WtE plants.

Moreover, WtE plants might generate local environmental benefits. The promise is that WtE eliminates the physical burden of waste, thereby reducing water and air pollution from landfills while at the same time, producing electricity (which is fed into the grid), hot water (for local heating or cooling systems), or steam (for the industry) [109]. In addition, they might help control the problems associated with illegal dumping and overflowing landfills, which cause air pollution, water pollution, and odor problems. Thus, we propose:

H3a: Public support for WtE facilities is higher when respondents perceive that their community is experiencing a problem with the disposal of plastic waste.
H3b: Public support for WtE facilities is higher when respondents perceive that their community is experiencing pollution and odor problems from landfills.
H3c: Public support for WtE facilities is higher when respondents perceive their community as benefiting from new jobs and investment opportunities.

## Data and methods

### Data

We administered the online survey to Thai respondents (18 years or older) using the services of the survey company, PureSpectrum. The survey was administered between February 22–26, 2024. The survey was originally composed in English, translated into the local Thai language by a native Thai speaker, and finally translated back into English to check for accuracy. Once our study was reviewed by the University of Washington’s human subjects committee, and consent was waived (#STUDY 00019824), we pre-registered our survey with OSF (https://osf.io/ebk7t/?view_only=e6572d7b6a6e458eb92466cc15eaee4c). At the start of our survey, we obtained written informed consent from survey participants.

### Survey design

Our survey was structured as follows. Participants first read the background information on plastic waste and WtE technology in Thailand. We then presented the pros and cons of WtE. We highlighted that while WtE plants can address multiple pollution problems from landfills and contribute to local economic development, they could create local air pollution and generate toxins that harm human health.

After noting the Thai government’s plans to construct new WtE plants, we randomly assigned respondents to three groups (the reference frame and two treatment frames). The first (reference), second, and third groups received information that WtE plants will be operated by local Thai companies (using local technology), Japanese (using Japanese technology), and Chinese companies (using Chinese technology), respectively (See Text A1 in the S1 Appendix for full texts of these frames).

We then asked respondents to indicate their support for a new WtE plant in their community on a 1–7 scale, our dependent variable. Specifically, we asked them to move the slider between 1 (Strongly oppose) and 7 (Strongly support) to indicate their level of support.

After indicating their level of support, respondents took an attention-check question. We asked each group about who would be operating the new plants according to the text that respondents had just read (which corresponds to the treatment information). While we initially administered the experiment to a sample of 2,000 respondents, 1,118 respondents failed to correctly answer the attention check question. Hence, we excluded them from our analysis. We also dropped respondents who selected the option, “Don’t know/ Prefer not to answer” (depending on the question) for at least one question used in our analysis. This yields our sample of 829. Given the concern that a high dropout rate might induce some biases, in Fig A1 in the S1 Appendix, we explored whether specific demographic groups selected incorrect answers (top) or received specific treatment frames (bottom) with the balance check tests. We found no such patterns. The values of the standardized biases for covariates (except ideology in the bottom figure) are well below 25, which is a cut point in social science [110]. As presented in Table A1 in the S1 Appendix, we also compared the demographic distribution between our sample and Thai census data by age and gender. While our sample of those who passed the attention check is comparable to the census data, it tends to overrepresent males in the age of 30–49 while underrepresenting females in the age of 18–29. As presented in Table A2 in the S1 Appendix, our main results hold even when we re-estimated the model with data weighted by age and gender to match the demographic distribution of the census.

The NIMBY (YIMBY) literature suggests that opposition to (support for) a local WtE plant could be influenced by risk perception about similar plants (Hypotheses 2) and different types of benefits (Hypotheses 3). Because a new WtE plant would create local air pollution, we posed a question about the pollution their community experiences from two other sources: coal-fired power plants and other existing waste incineration. Thus, we asked about respondents’ agreement/disagreement with the statements: “My local community experiences air pollution from waste incineration” (*Incineration*) and “My local community experiences air pollution from coal-fired power plants” (*Coal*), respectively.

WtEs create local environmental and economic benefits. Arguably, perceptions about pollution from municipal waste or existing landfills might inform support for a future WtE plant. To assess perceptions WtE’s environmental benefits (emanating from reducing the pressure on landfills that cause local water and air pollution problems), we asked respondents’ agreement/disagreement with the statements: “My local community experiences the plastic waste problem” (*Waste*) and “My local community experiences odor, noise, or hygiene issues due to soil and water pollution from landfills.” (*Landfill*), respectively.

YIMBY literature suggests that communities might support “dirty” facilities that create local jobs. To gauge the perception of economic benefit, we asked about WtE plants’ contribution to local economic development by creating new jobs and investment opportunities (*Job*). The underlying perceptions about the seriousness of pollution problems could independently influence the level of support for WtE plants. Hence, we asked about support for the Thai government’s recent ban on the manufacturing and sale of single-use plastics such as plastic bags, plastic utensils, and plastic containers (*Plastic*).

Global South countries like Thailand are characterized by a high level of corruption. In 2023, Thailand was ranked 108 out of 180 countries by Transparency International’s *Global Corruption Perception Index* [111]. Scholars note that corruption is associated with poor enforcement of environmental laws [112]. WtEs are subject to several environmental laws given their impact on local air and water pollution. Thus, we asked about respondents’ agreement with the statement “Most local factories in Thailand do not follow environmental laws.” (*Corrupt*).

Toward the end of the survey, we included standard demographic questions on gender (*Gender*), age (*Age*), education (*Education*), income (*Income*), and political ideology (*Ideology*).

### Model

To test our hypotheses, we constructed the following OLS model. Our dependent variable (*Y*), support for the construction of WtE plants in the local community, ranges between 1 and 7 (on a slider scale and, therefore, is continuous). *Japan* and *China* represent treatment frames. In addition, our main variables include risk perception (*Incineration* and *Coal*) and perception of environmental and economic benefits (*Waste, Landfill*, and *Job*). In addition, we control for support for the plastic ban (*Plastic*) and that facilities obey environmental laws (*Corruption*). Demographic variables are *Age, Gender, Income, Education*, and *Ideology*.


$$\hat{Y_{i}}=\alpha +\beta_{1}{Treatment}_{1,i}+{\beta_{2}{Incineration}_{i}+{\beta_{3}Coal}_{i}+\;\beta }_{4}{Waste}_{3,i}+{\beta_{5}Landfill}_{i}\;+\;{\beta_{6}Job}_{i}+\;{\beta_{7}Plastic}_{i}+\;{\beta_{8}Corrupt}_{i}+\;\gamma^{\prime }X_{i}$$

where $X_{i}$ represents a vector of *Age, Gender, Education, Income, and Ideology.*

## Results

**Fig 1:**
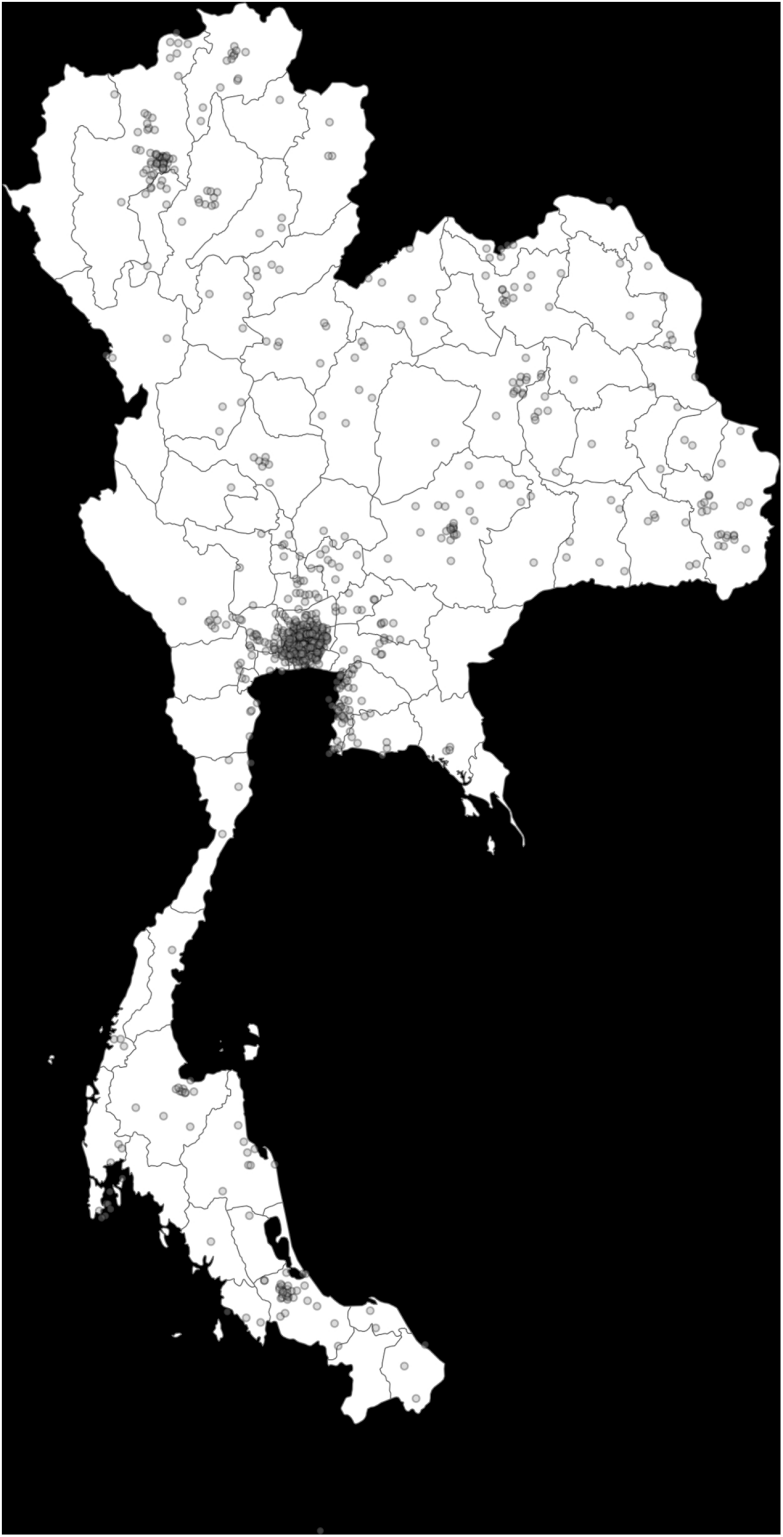
Geographical distribution of respondents of our sample.

Table 1 presents the descriptive statistics for the variables used in our analyses. In Fig 1, the geographical distribution of respondents based on their zip-code information are presented (Fig 1). The geographical locations of inattentive respondents and WtE plants, landfills, and coal-fired power plants is respectively presented in Figs A2 and A3 in the S1 Appendix. Maps in Fig A2 shows that the geographical distribution between attentive and inattentive respondents are comparable. Our balance check also confirmed that the distance between respondents and existing waste power plants, landfills, and a coal-fired power plant is not statistically significantly different between the attentive and inattentive respondents

**Table 1:** Descriptive statistics.

Variable	Mean	SD	Min	Max
Outcome	5.344	1.551	1	7
Control (Thailand)	0.449	0.498	0	1
Treatment (Japan)	0.317	0.466	0	1
Treatment (China)	0.234	0.424	0	1
Incineration	3.539	1.212	1	5
Coal	3.072	1.270	1	5
Waste	3.836	1.023	1	5
Landfill	3.439	1.206	1	5
Job	3.952	0.876	1	5
Plastic	5.764	1.330	1	7
Corruption	3.806	1.024	1	5
Age	43.667	15.515	18	110
Gender (Male)	0.468	0.499	0	1
Gender (Female)	0.528	0.499	0	1
Gender (Non-binary)	0.004	0.060	0	1
Education	5.181	1.467	1	7
Income	5.463	2.241	1	9
Ideology	3.332	1.380	1	5

The mean value of the dependent variable is 5.3 on 1–7 scale, suggesting that respondents are quite supportive of new WtE plants. This support level is surprising because the media widely reports on local opposition to WtE plants. Arguably, the relatively high support levels suggest that respondents perceive WtE’s high levels of benefits or are not worried about the local risks posed by these plants.

### Main results

As shown in Table 2, with *Thai* as the reference category, *China* has a statistically significant negative effect on WtE support (Hypothesis 1b is supported), while the effect of *Japan* is not statistically significant (H1a is not supported). This might reflect that Thai respondents associate Chinese Technology and firms with negative environmental impacts. We were surprised to see the lack of significance for Japanese firms and technologies because Japanese companies tend to have strong reputations for their environmental technology [113,114] and their respect for local laws.

**Table 2:** Main result.

(Intercept)	1.597	(0.360)	^***^
Japan	−0.033	(0.104)	
China	−0.528	(0.113)	^***^
Incineration	0.011	(0.058)	
Coal	0.182	(0.047)	^***^
Waste	−0.048	(0.057)	
Landfill	−0.052	(0.055)	
Job	0.650	(0.056)	^***^
Plastic	0.320	(0.036)	^***^
Corruption	−0.168	(0.047)	^***^
Age	0.002	(0.003)	
Gender: female	−0.101	(0.094)	
Gender: non-binary	−1.044	(0.744)	
Education	−0.072	(0.032)	^*^
Income	0.063	(0.022)	^**^
Ideology	−0.064	(0.034)	
N	829		
AIC	2764.9		
RMSE	1.26		

*Note*: Cluster-robust standard errors in parentheses; * p < 0.05, ** p < 0.01, *** p < 0.001

Regarding factors driving risk perception from existing air pollution sources, *Coal* has a statistically significant positive effect on public support for WtE plants (Hypothesis 2b is supported) while *Incineration is* not statistically significant (Hypothesis 2a is not supported). Thus, those who perceive a high risk of air pollution from coal plants are *more* supportive of WtE plants. One plausible explanation is that respondents view WtE plants to be cleaner than coal-fired electricity plants that they plausibly replace. Or, even if WtE does not replace existing coal plants, WtE might reduce dependence on coal-fired plants in the future. Communities near coal-fired electricity plants are affected by coal dust and other pollutants. For example, sulfur dioxide exposure from Mae Moh Coal Power Plant, especially among children, led residents to file lawsuits for compensation for death and health due to respiratory diseases [115]. In 2017, to end widespread conflicts over the construction of coal-fired power plants, the Prime Minister also instructed the Ministry of Natural Resources and Environment to conduct new environmental and health impact assessments and requested public hearings with all stakeholders [115].

Among the variables related to perceptions of local environmental and economic benefits (*Waste, Landfill*, and *Job*), only *Job* has a statistically significant positive effect (H3c is supported). However, the environmental benefits of waste and landfill reduction do not seem to influence support for WtE facilities (Hypotheses 3a and 3b are not supported).

*Plastic* and *Corruption,* respectively have a statistically significant effect, as we expected. Thus, respondents supporting the plastic ban are more supportive of WtE plants. In addition, those who are distrustful of the legal compliance of local factories are less supportive of WtE plants.

Among demographic variables, *Education* and *Income,* respectively have a statistically significant effect in the expected direction, while *Age, Gender*, and *Ideology* have no such effect.

## Conclusion

Scholars have viewed local support for “dirty” projects primarily through the NIMBY lens. The intuition is that communities do not want to bear local costs imposed by projects that produce public goods whose benefits spill over to other jurisdictions. Our paper suggests that even for “dirty” projects, NIMBY and YIMBY might be working together. This is because some “dirty” projects, while increasing local environmental and health risks, might also create local environmental and economic benefits. We can extend our analytical framework to assess local opposition to treatment facilities or landfill sites for other types of waste, such as food waste, which is another critical challenge in Thailand [116].

Second, much of the NIMBY/YIMBY work has focused on how project characteristics shape local perceptions about its benefits and costs. In our paper, holding the project type (WtE) constant, we varied operator characteristics. Our findings suggest that benefit/cost perceptions might be shaped by operator characteristics (national origin in our case), an issue that future work should explore. With globalization, multinational corporations are increasingly involved in projects that pose local risks and also generate benefits. As the “country-of-origin” and the “liability of foreignness” literature notes, local communities might oppose foreign projects. However, multinationals could come from Western countries as well as Asian countries such as Japan, China, and India. Thus, local communities might assess the benefits/costs of projects based on their perceptions of the countries of origin of multinational corporations.

Further, our paper finds that only Chinese companies face the “penalty of foreignness,” not Japanese companies. At the same time, Japanese companies do not enjoy the “premium for foreignness” either, notwithstanding the positive perceptions about their compliance with environmental laws and reliable technology. The antipathy towards Chinese operators is puzzling because Thai citizens tend to be generally supportive of China. A 2024 Pew Survey finds that 80% of Thai respondents expressed favorable views towards China, compared to 12% in Japan and 16% in the US [117]. Similarly, a 2022 public opinion survey administered by the Central European Institute of Asian Studies finds that 80% of Thai respondents feel positive or very positive toward China, which is the third highest among Asia & Pacific countries after Pakistan and Bangladesh [118]. So, why the distrust of Chinese WtE plants?

We speculate that while Thai people have positive attitudes toward China, they are cautious when China operates infrastructure projects that pose local environmental and health risks. Despite some evidence of the positive local economic impact of China’s foreign investment projects [119], broadly, there is ample evidence of local backlash in African countries against Chinese projects [120–122] and the rise of anti-Chinese populism [57]. This is rooted in the perception that Chinese companies have minimal local economic impact because they tend to bring along Chinese nationals, as opposed to employing the local population (but also see [123]). In addition, the post-COVID debt crisis suggested that Chinese BRI funding for prestige projects [124] has increased the debt burden on some countries in the Global South. Chinese infrastructure projects might be viewed less favorably for this reason. Future work should explore local assessments of infrastructure projects that are managed by companies from different countries.

Our paper has several limitations. First, we focused on a specific type of infrastructure/energy project, WtE, which has specific risk/benefit characteristics. Future work should assess public perceptions of projects with different risk-benefit profiles. Second, much to our surprise, about 55% of the respondents did not pass the comprehension check, although before the main text, we inserted instructions strongly encouraging them to read carefully and informally pre-tested the survey instrument with native speakers. We believe there is an opportunity to sharpen the instrument to ensure that a higher share of respondents respond to surveys with more attention and higher comprehension.

## Supporting information

S1 Appendix Penalty or premium for foreign ownership in Thailand ? Comparing public support for waste-to-energy plants operated by Thai, Japanese, and Chinese Companies.
(DOCX)

## References

[1] Simon HA. Models of bounded rationality: Empirically grounded economic reason. Vol 3. Cambridge (MA): MIT Press; 1997.

[2] Johnson TR. Regulatory dynamism of environmental mobilization in urban China. Regul Gov. 2016;10(1):14–28.

[3] Baxter J, Ho Y, Rollins Y, Maclaren V. Attitudes toward waste to energy facilities and impacts on diversion in Ontario, Canada. Waste Manag. 2016;50:75–85. doi: 10.1016/j.wasman.2016.02.017 26951720

[4] Collingsworth T, Goold JW, Harvey PJ. Time for a global new deal. Foreign Affairs. 1994;73(1):8. doi: 10.2307/20045886

[5] Gill S. Globalisation, market civilisation, and disciplinary neoliberalism. Millennium: J Int Stud. 1995;24(3):399–423. doi: 10.1177/03058298950240030801

[6] Leonard HJ. Pollution and the struggle for the world product. Cambridge: Cambridge University Press; 1988.

[7] Charnovitz S. Environmentalism confronts GATT rules. J World Trade. 1993;27(2):37–52.

[8] Daly H. The perils of free trade. Sci Am. 1993;269(5):51–5.

[9] Jaffe AB, Peterson SL, Portney PR, Stavins RN. Environmental regulation and the competitiveness of U.S. manufacturing: what does the evidence tell us? J Econ Lit. 1995;33(1):132–63.

[10] Mani M, Wheeler D. In search of pollution havens? Why do firms move their polluting plants to developing countries? J Environ Dev. 1998;7(3):215–47.

[11] Bai Y, Givens J. Ecologically unequal exchange of plastic waste? A longitudinal analysis of international trade in plastic waste. J World Syst Res. 2021;27(1):265–87.

[12] Liu X, Lei T, Boré A, Lou Z, Abdouraman B, Ma W. Evolution of global plastic waste trade flows from 2000 to 2020 and its predicted trade sinks in 2030. J Clean Prod. 2022;376:134373.

[13] Zaheer S. Overcoming the liability of foreignness. Acad Manage J. 1995;38(2):341–63.

[14] Zhou N, Guillén MF. From home country to home base: a dynamic approach to the liability of foreignness. Strateg Manage J. 2015;36(6):907–17.

[15] Cao M, Alon I. Overcoming the liability of foreignness – a new perspective on Chinese MNCs. J Bus Res. 2021;128:611–26. doi: 10.1016/j.jbusres.2020.11.017

[16] Gunningham N, Kagan RA, Thornton D. Social license and environmental protection: why businesses go beyond compliance. Law Soc Inq. 2004;29(2):307–41. doi: 10.1111/j.1747-4469.2004.tb00338.x

[17] Demuijnck G, Fasterling B. The social license to operate. J Bus Ethics. 2016;136(4):675–85. doi: 10.1007/s10551-015-2976-7

[18] Porter ME. The competitive advantage of nations. New York: Free Press; 1990.

[19] Sethi SP, Elango B. The influence of “country of origin” on multinational corporation global strategy: a conceptual framework. J Int Manag. 1999;5(4):285–98. doi: 10.1016/s1075-4253(99)00016-2

[20] Van Tulder R, Kolk A. Multinationality and corporate ethics. J Int Bus Stud. 2001;32(2):267–83.

[21] Pauly LW, Reich S. National structures and multinational corporate behavior: enduring differences in the age of globalization. Int Org. 1997;51(1):1–30. doi: 10.1162/002081897550285

[22] Prakash A, Potoski M. Investing up: FDI and the cross-country diffusion of ISO 14001 management systems. Int Stud Q. 2007;51(3):723–44.

[23] Moeller M, Harvey M, Griffith D, Richey G. The impact of country-of-origin on the acceptance of foreign subsidiaries in host countries: an examination of the ‘liability-of-foreignness. Int Bus Rev. 2013;22(1):89–99.

[24] Dear M. Understanding and overcoming the NIMBY syndrome. J Am Plan Assoc. 1992;58(3):288–300.

[25] Wolsink M. Invalid theory impedes our understanding: a critique on the persistence of the language of NIMBY. Trans Inst Br Geogr. 2006;31(1):85–91.

[26] Bullard RD. Dumping in Dixie: Race, class, and environmental quality. New York: Routledge; 2018.

[27] Groothuis PA, Miller G. Locating hazardous waste facilities: the influence of NIMBY beliefs. Am J Econ Sociol. 1994;53(3):335–46.

[28] Hunter S, Leyden KM. Beyond NIMBY: explaining opposition to hazardous waste facilities. Policy Stud J. 1995;23(4):601–19.

[29] McGurty EM. From NIMBY to civil rights: the origins of the environmental justice movement. Environ Hist. 1997;2(3):301–23.

[30] Schively C. Understanding the NIMBY and LULU phenomena: reassessing our knowledge base and informing future research. J Plan Lit. 2007;21(3):255–66.

[31] Whittemore AH, BenDor TK. Reassessing NIMBY: the demographics, politics, and geography of opposition to high-density residential infill. J Urban Aff. 2018;41(4):423–42. doi: 10.1080/07352166.2018.1484255

[32] Pollution Control Department. Ministry of Natural Resources and Environment. Action Plan on Plastic Waste Management Phase 2 (2023-2027) [Internet]. 2023 [cited 2025 Jun 1]. Available from: https://www.pcd.go.th/wp-content/uploads/2023/06/pcdnew-2023-06-15_08-07-42_392659.pdf

[33] Fegade SL. Green hydrocarbons and fuels from municipal polymer waste co-fed with natural gas using a batch catalytic slurry process. Green Technol Sustain. 2024;2(3):100099. doi: 10.1016/j.grets.2024.100099

[34] Tantivangphaisal P. Sustainable solutions: Bangkok’s waste-to-energy plants [Internet]. Thaiger; 2024 [cited 2025 Jun 1]. Available from: https://thethaiger.com/news/national/sustainable-solutions-bangkoks-waste-to-energy-plants

[35] Wachpanich N, Coca N. As waste-to-energy incinerators spread in Southeast Asia, so do concerns [Internet]. Mongabay; 2022 [cited 2025 Jun 1]. Available from: https://news.mongabay.com/2022/12/as-waste-to-energy-incinerators-spread-in-southeast-asia-so-do-concerns/

[36] Bangkok Business. Why are “waste power plants” opposed almost everywhere? [Internet]. Bangkok Business News; 2022 [cited 2025 Jun 1]. Available from: https://www.bangkokbiznews.com/social/1000904

[37] Manager Online. Anti-Korat Waste Power Plant Heats Up! Mobs from 2 Districts Set Up Camp in Front of City Hall Until Project Cancellation [Internet]. 2023 [cited 2025 Jun 1]. Available from: https://mgronline.com/local/detail/9660000071946

[38] Jiang Y. Competitive partners in development financing: China and Japan expanding overseas infrastructure investment. Pac Rev. 2019;32(5):778–808.

[39] Yamamoto R. China’s development assistance in Southeast Asia: a threat to Japanese interests? Asian Surv. 2020;60(2):323–46.

[40] Liao JC, Katada SN. Institutions, ideation, and diffusion of Japan’s and China’s overseas infrastructure promotion policies. New Polit Econ. 2021;27(6):944–57. doi: 10.1080/13563467.2021.1961219

[41] Kyodo News. Japan, Thailand to launch new energy initiative for decarbonization [Internet]. 2022 [cited 2025 Jun 1]. Available from: https://english.kyodonews.net/news/2022/01/d904f55df2e7-japan-thailand-to-launch-new-energy-initiative-for-decarbonization.html

[42] Pike L. Green belt and road [Internet]. The Spotlight - China Dialogue; 2019 [cited 2025 Jun 1]. Available from: https://chinadialogue.net/en/energy/11212-green-belt-and-road-in-thespotlight/

[43] Marston H. Bauxite Mining in Vietnam’s central highlands: an arena for expanding civil society? Contemp Southeast Asia. 2012;34(2):173. doi: 10.1355/cs34-2b

[44] Tracy EF, Shvarts E, Simonov E, Babenko M. China’s new Eurasian ambitions: the environmental risks of the silk road economic belt. Eurasian Geogr Econ. 2017;58(1):56–88. doi: 10.1080/15387216.2017.1295876

[45] Mori A. Foreign actors, faster transitions? Co-evolution of complementarities, perspectives and sociotechnical systems in the case of Indonesia’s electricity supply system. Energy Res Soc Sci. 2020;69:101594. doi: 10.1016/j.erss.2020.101594

[46] Tritto A. China’s belt and road initiative: from perceptions to realities in Indonesia’s coal power sector. Energy Strategy Rev. 2021;34:100624. doi: 10.1016/j.esr.2021.100624

[47] The Economist. Global firms are eyeing Asian alternatives to Chinese manufacturing [Internet]. 2023 [cited 2025 Jun 1]. Available from: https://www.economist.com/business/2023/02/20/global-firms-are-eyeing-asian-alternatives-to-chinese-manufacturing

[48] Chong YT, Teo KM, Tang LC. A lifecycle-based sustainability indicator framework for waste-to-energy systems and a proposed metric of sustainability. Renew Sust Energ Rev. 2016;56:797–809.

[49] Kumar A, Samadder SR. A review on technological options of waste to energy for effective management of municipal solid waste. Waste Manag. 2017;69:407–22. doi: 10.1016/j.wasman.2017.08.046 28886975

[50] Abd Kadir SAS, Yin C-Y, Rosli Sulaiman M, Chen X, El-Harbawi M. Incineration of municipal solid waste in Malaysia: salient issues, policies and waste-to-energy initiatives. Renew Sustain Energy Rev. 2013;24:181–6. doi: 10.1016/j.rser.2013.03.041

[51] Ali S, Ahmed W, Solangi YA, Chaudhry IS, Zarei N. Strategic analysis of single-use plastic ban policy for environmental sustainability: the case of Pakistan. Clean Technol Environ Policy. 2022;1–7.36536780

[52] Deutsche Welle. European firms help Southeast Asia turn trash into energy [Internet]. 2023 [cited 2025 Jun 1]. Available from: https://www.dw.com/en/european-firms-help-southeast-asia-turn-trash-into-energy/a-64325843

[53] Energy Monitor. Facing headwinds at home. Europe and Japan are pushing waste-to-energy technology across South East Asia [Internet]. 2022 [cited 2025 Jun 1]. Available from: https://www.energymonitor.ai/sectors/industry/facing-headwinds-at-home-europe-and-japan-are-pushing-waste-to-energy-technology-across-south-east-asia/

[54] Li Y, Shapiro J. China goes green: coercive environmentalism for a troubled planet. Cambridge: Polity Press; 2020.

[55] Negi R. Beyond the “Chinese scramble”: the political economy of anti-China sentiment in Zambia. Afr Geogr Rev. 2008;27(1):41–63.

[56] Hess S, Aidoo R. Charting the roots of anti-Chinese populism in Africa. Cham: Springer International Publishing; 2015.

[57] Sibiri H. The emerging phenomenon of anti-Chinese populism in Africa: Evidence from Zambia, Zimbabwe and Ghana. Insight Afr. 2021;13(1):7–27.

[58] Urdinez F. “They own our country!” voter reaction to anti-China rhetoric: The case of the presidential election in Brazil in 2018. Electoral Stud. 2023;86:102708. doi: 10.1016/j.electstud.2023.102708

[59] Adolph C, Quince V, Prakash A. The Shanghai effect: do exports to China affect labor practices in Africa? World Dev. 2017;89:1–18.

[60] Yoshimatsu H. Japan’s strategic response to China’s geo-economic presence: quality infrastructure as a diplomatic tool. Pac Rev. 2023;36(1):148–76.

[61] Russel DR, Berger B. Navigating the belt and road initiative [Internet]. A report of the Asia Society Policy Institute; 2019 [cited 2025 Jun 1]. Available from: https://asiasociety.org/sites/default/files/2019-06/Navigating%20the%20Belt%20and%20Road%20Initiative_2.pdf

[62] Naím M. Rogue aid. Foreign Policy. 2007;159:95–6.

[63] Woods N. Whose aid? Whose influence? China, emerging donors and the silent revolution in development assistance. Int Aff. 2008;84(6):1205–12121.

[64] Saha S. The climate risks of China’s belt and road initiative. Bull At Sci. 2020;76(5):249–55.

[65] Ministry of Foreign Affairs of Japan. Quality Infrastructure [Internet]. 2023 [cited 2025 Jun 1]. Available from: https://www.mofa.go.jp/policy/oda/sector/infrastructure/index.html

[66] Ekmekçioğlu M, Kaya T, Kahraman C. Fuzzy multicriteria disposal method and site selection for municipal solid waste. Waste Manag. 2010;30(8–9):1729–36. doi: 10.1016/j.wasman.2010.02.031 20303733

[67] Song J, Song D, Zhang X, Sun Y. Risk identification for PPP waste-to-energy incineration projects in China. Energy Policy. 2013;61:953–62. doi: 10.1016/j.enpol.2013.06.041

[68] Ghoseiri K, Lessan J. Waste disposal site selection using an analytic hierarchal pairwise comparison and ELECTRE approaches under fuzzy environment. J Intell Fuzzy Syst. 2014;26(2):693–704. doi: 10.3233/ifs-120760

[69] Wang L, Zhang X. Bayesian analytics for estimating risk probability in PPP waste-to-energy projects. J Manag Eng. 2019;34(6):04018047.

[70] Jenkins-Smith HC, Silva CL, Nowlin MC, deLozier G. Reversing nuclear opposition: evolving public acceptance of a permanent nuclear waste disposal facility. Risk Anal. 2011;31(4):629–44. doi: 10.1111/j.1539-6924.2010.01543.x 21175714

[71] Sherman DJ. Not here, not there, not anywhere: politics, social movements, and the disposal of low-level radioactive waste. New York: Routledge; 2012.

[72] Lober DJ, Green DP. NIMBY or NIABY: a logit model of opposition to solid-waste-disposal facility siting. J Environ Manage. 1994;40(1):33–50.

[73] Portney KE. Allaying the NIMBY syndrome: the potential for compensation in hazardous waste treatment facility siting. Hazard Waste. 1984;1(3):411–21. doi: 10.1089/hzw.1984.1.411

[74] Swofford J, Slattery M. Public attitudes of wind energy in Texas: local communities in close proximity to wind farms and their effect on decision-making. Energy Policy. 2010;38(5):2508–19. doi: 10.1016/j.enpol.2009.12.046

[75] Ek K. Public and private attitudes towards “green” electricity: the case of Swedish wind power. Energy Policy. 2005;33(13):1677–89. doi: 10.1016/j.enpol.2004.02.005

[76] Stokes LC. Electoral backlash against climate policy: a natural experiment on retrospective voting and local resistance to public policy. Am J Pol Sci. 2016;60(4):958–74.

[77] Ko I, Dolšak N, Prakash A. Wind turbines as new smokestacks: Preserving ruralness and restrictive land-use ordinances across U.S. counties. PLoS One. 2023;18(12):e0294563. doi: 10.1371/journal.pone.0294563 38091303 PMC10718419

[78] Devine‐Wright P. Public engagement with large‐scale renewable energy technologies: breaking the cycle of NIMBYism. WIREs Clim Change. 2010;2(1):19–26. doi: 10.1002/wcc.89

[79] Kim Y, Kim M, Kim W. Effect of the Fukushima nuclear disaster on global public acceptance of nuclear energy. Energy Policy. 2013;61:822–8. doi: 10.1016/j.enpol.2013.06.107

[80] Gamson W, Modigliani A. Media discourse and public opinion on nuclear power: a constructionist approach. Am J Sociol. 1989;95(1):1–37.

[81] Kemp R. Why not in my backyard? A radical interpretation of public opposition to the deep disposal of radioactive waste in the United Kingdom. Environ Plan A. 1990;22(9):1239–58.

[82] He G, Mol A, Zhang L, Lu Y. Public participation and trust in nuclear power development in China. Renew Sust Energ Rev. 2013;23:1–11.

[83] Coval JD, Moskowitz TJ. Home bias at home: local equity preference in domestic portfolios. J Fin. 1999;54(6):2045–73.

[84] Garmaise MJ, Moskowitz TJ. Confronting information asymmetries: evidence from real estate markets. Rev Financ Stud. 2004;17(2):405–37.

[85] Fatima N, Li Y, Ahmad M, Jabeen G, Li X. Factors influencing renewable energy generation development: a way to environmental sustainability. Environ Sci Pollut Res Int. 2021;28(37):51714–32. doi: 10.1007/s11356-021-14256-z 33988841

[86] Yasmeen H, Tan Q, Ali S, Ismail H. Managing environmental quality in Pakistan through sustainable development of energy–economy–environment (3E): insights from graph model of conflict resolution (GMCR). Manag Environ Qual. 2021;32(5):1095–111.

[87] Song L, Sun Y, Song J, Feng Z, Gao J, Yao Q. From “not in my backyard” to “please in my backyard”: transforming the local responses toward a waste-to-energy incineration project in China. Sustain Prod Consum. 2024;45:104–14.

[88] Kikuchi R, Gerardo R. More than a decade of conflict between hazardous waste management and public resistance: a case study of NIMBY syndrome in Souselas (Portugal). J Hazard Mater. 2009;172(2–3):1681–5. doi: 10.1016/j.jhazmat.2009.07.062 19679393

[89] Sun C, Ouyang X, Meng X. Public acceptance towards waste-to-energy power plants: a new quantified assessment based on “willingness to pay”. J Environ Plan Manage. 2019;62(14):2459–77.

[90] Lu J-W, Xie Y, Xu B, Huang Y, Hai J, Zhang J. From NIMBY to BIMBY: An evaluation of aesthetic appearance and social sustainability of MSW incineration plants in China. Waste Manag. 2019;95:325–33. doi: 10.1016/j.wasman.2019.06.016 31351619

[91] Achillas C, Vlachokostas C, Moussiopoulos N, Banias G, Kafetzopoulos G, Karagiannidis A. Social acceptance for the development of a waste-to-energy plant in an urban area. Resour Conserv Recycl. 2011;55(9–10):857–63.

[92] Davies AR. Civil society activism and waste management in Ireland: the Carranstown anti-incineration campaign. Land Use Policy. 2008;25(2):161–72. doi: 10.1016/j.landusepol.2007.04.002

[93] Caferra R, D’Adamo I, Morone P. Wasting energy or energizing waste? The public acceptance of waste-to-energy technology. Energy. 2023;263:126123. doi: 10.1016/j.energy.2022.126123

[94] Woo A, Joh K, Yu C-Y. Who believes and why they believe: individual perception of public housing and housing price depreciation. Cities. 2021;109:103019. doi: 10.1016/j.cities.2020.103019

[95] Cong X, Wang L, Ma L, Skibnewski M. Exploring critical influencing factors for the site selection failure of waste-to-energy projects in China caused by the “not in my back yard” effect. Eng Constr Archit Manag. 2020;28(6):1561–92. doi: 10.1108/ecam-12-2019-0709

[96] Zhou Q, Xu M, Liu Y, Cui C, Xia B, Ke Y, et al. Exploring the effects of spatial distance on public perception of waste-to-energy incineration projects. Waste Manag. 2022;143:168–76. doi: 10.1016/j.wasman.2022.02.033 35263667

[97] Coca N. Coal on the Javan Coast [Internet]. Hakai Magazine; 2019 [cited 2025 Jun 1]. Available from: www.hakaimagazine.com/news/coal-on-the-javan-coast/

[98] Wijaya T. Conditioning a stable sustainability fix of ‘ungreen’ infrastructure in Indonesia: transnational alliances, compromise, and state’s strategic selectivity. The Pac Rev. 2021;35(5):821–52. doi: 10.1080/09512748.2021.1884123

[99] Lim G. China-Japan Rivalry and Southeast Asian renewable energy development: who is winning what in Indonesia? Asian Perspect. 2022;46(1):105–32.

[100] Thomas A. Why coal-fired power plants in Southeast Asia are facing opposition heat [Internet]. Down to Earth; 2021 [cited 2025 Jun 1]. Available from: https://www.downtoearth.org.in/environment/why-coal-fired-power-plants-in-southeast-asia-are-facing-opposition-heat-75348

[101] Warren CR, Lumsden C, O’Dowd S, Birnie RV. Green on green: public perceptions of wind power in Scotland and Ireland. J Environ Plan Manage. 2005;48(6):853–75.

[102] Greenberg MR. NIMBY, CLAMP, and the location of new nuclear-related facilities: U.S. national and 11 site-specific surveys. Risk Anal. 2009;29(9):1242–54. doi: 10.1111/j.1539-6924.2009.01262.x 19572962

[103] Gravelle TB, Lachapelle E. Politics, proximity and the pipeline: mapping public attitudes toward Keystone XL. Energy Policy. 2015;83:99–108.

[104] Clarke CE, Bugden D, Hart PS, Stedman RC, Jacquet JB, Evensen DT. How geographic distance and political ideology interact to influence public perception of unconventional oil/natural gas development. Energy Policy. 2016;97:301–9.

[105] Culley MR, Ogley‐Oliver E, Carton AD, Street JC. Media framing of proposed nuclear reactors: an analysis of print media. J Community Appl Soc Psychol. 2010;20(6):497–512. doi: 10.1002/casp.1056

[106] Venables D, Pidgeon NF, Parkhill KA, Henwood KL, Simmons P. Living with nuclear power: sense of place, proximity, and risk perceptions in local host communities. J Environ Psychol. 2012;32(4):371–83.

[107] Uji A, Prakash A, Song J. Does the “NIMBY syndrome” undermine public support for nuclear power in Japan? Energy Policy. 2021;148:111944. doi: 10.1016/j.enpol.2020.111944

[108] Thorpe R. Perverse politics: the persistence of mass imprisonment in the twenty-first century. Perspect Polit. 2015;13(3):618–37.

[109] Yan M, P A, Waluyo J. Challenges for sustainable development of waste to energy in developing countries. Waste Manag Res. 2020;38(3):229–31. doi: 10.1177/0734242X20903564 32054434

[110] Ho D, Imai K, King G, Stuart EA. MatchIt: nonparametric preprocessing for parametric causal inference. J Stat Softw. 2011;42:1–28.

[111] Transparency International. Corruption Perceptions Index 2024 [Internet]. 2024 [cited 2025 Jun 1]. Available from: https://www.transparency.org/en/countries/thailand

[112] Berliner D, Prakash A. Signaling environmental stewardship in the shadow of weak governance: the global diffusion of ISO 14001. Law Soc Rev. 2013;47(2):345–73.

[113] Gutowski T, Murphy C, Allen D, Bauer D, Bras B, Piwonka T, et al. Environmentally benign manufacturing: observations from Japan, Europe and the United States. J Clean Prod. 2005;13(1):1–17. doi: 10.1016/j.jclepro.2003.10.004

[114] Endo K. Corporate governance beyond the shareholder–stakeholder dichotomy: lessons from Japanese corporations’ environmental performance. Bus Strat Env. 2020;29(4):1625–33. doi: 10.1002/bse.2457

[115] Komchadluek. How many “coal-fired power plants” does Thailand have? [Internet]. Komchadluek; 2017 [cited 2025 Jun 1]. Available from: https://www.komchadluek.net/news/scoop/270838

[116] Srijuntrapun P, Ket-Um P, Attavanich W. Socio-demographic drivers of household food waste management practices in Thailand. PLoS One. 2025;20(4):e0321054. doi: 10.1371/journal.pone.0321054 40168395 PMC11960981

[117] Bangkok Business. ‘Thailand’ sees ‘China’ in the most positive light in the world, survey results lead 80%, the only country [Internet]. Bangkok Business News; 2024 [cited 2025 Jun 1]. Available from: https://www.bangkokbiznews.com/world/1135197

[118] Turcsányi RQ, Kironská K, Gerstl A, Dubravčíková K, Iocovozzi J, Gries P, et al. Public opinion in the Indo-Pacific: Divided on China, cheering for US & EU [Internet]. Central European Institute of Asian Studies (CEIAS); 2022 [cited 2025 Jun 1]. Available from: https://ceias.eu/wp-content/uploads/2022/11/Draft-2_FINAL.pdf

[119] Bunte JB, Desai H, Gbala K, Parks B, Runfola DM. Natural resource sector FDI, government policy, and economic growth: quasi-experimental evidence from Liberia. World Dev. 2018;107:151–62.

[120] Obi CI. Enter the dragon? Chinese oil companies & resistance in the Niger Delta. Rev Afr Polit Econ. 2008;35(117):417–34.

[121] Sautman B, Hairong Y. African perspectives on China–Africa Links. China Q. 2009;199:728–59. doi: 10.1017/s030574100999018x

[122] Zhao S. A neo-colonialist predator or development partner? China’s engagement and rebalance in Africa. J Contemp China. 2014;23(90):1033–52. doi: 10.1080/10670564.2014.898893

[123] Parks B, Bluhm R, Dreher A, Fuchs A, Strange AM, Tierney MJ. Belt and Road Projects Direct Chinese Investment to All Corners of the Globe. What Are the Local Impacts? [Internet]. Monkey Cage - The Washington Post; 2018 [cited 2025 Jun 2]. Available from: https://www.washingtonpost.com/news/monkey-cage/wp/2018/09/11/belt-and-road-projects-direct-chinese-investment-to-all-corners-of-the-globe-what-are-the-local-impacts/

[124] Hurley J, Morris S, Portelance G. Examining the debt implications of the Belt and Road Initiative from a policy perspective. J Infrastruct Policy Dev. 2019;3(1):139–75.

