# Supplementary material for: Penalty or premium for foreign ownership in Thailand? Comparing public support for waste-to-energy plants operated by Thai, Japanese, and Chinese Companies
Source: PLoS One. 2025 Jul 10;20(7):e0328165. doi: 10.1371/journal.pone.0328165 (PMC12244724; doi:10.1371/journal.pone.0328165)
Supplement: S1 Appendix — ? Comparing public support for waste-to-energy plants operated by Thai, Japanese, and Chinese Companies. (DOCX) [file pone.0328165.s001.docx]

***Online Appendix***

***for***

***“Penalty or Premium for Foreign Ownership in Thailand?***

***Comparing Public Support for Waste-to-Energy Plants Operated by Thai, Japanese, and Chinese Companies”***

**Table of Contents**

**Figure A1.** Balance check

**Figure A2.** Geographical distribution of inattentive respondents

**Figure A3.** Geographical location of coal-fired power plants, landfills, and waste incineration facilities

**Table A1.** Comparison of demographic distribution between our sample and the census data by age and gender

**Table A2.** Results based on the weighted data

**Text A1.** Full text of survey questions

**Figure A1. Balance check**

Balance between those who passed the attention check and those who did not


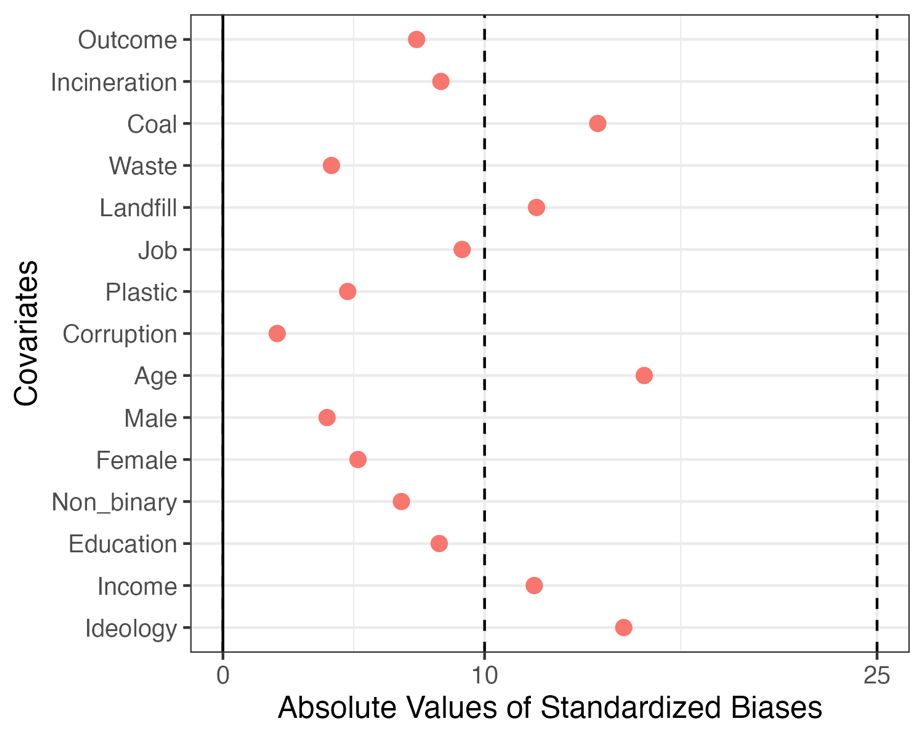


Balance across treatment groups among those who passed the attention check


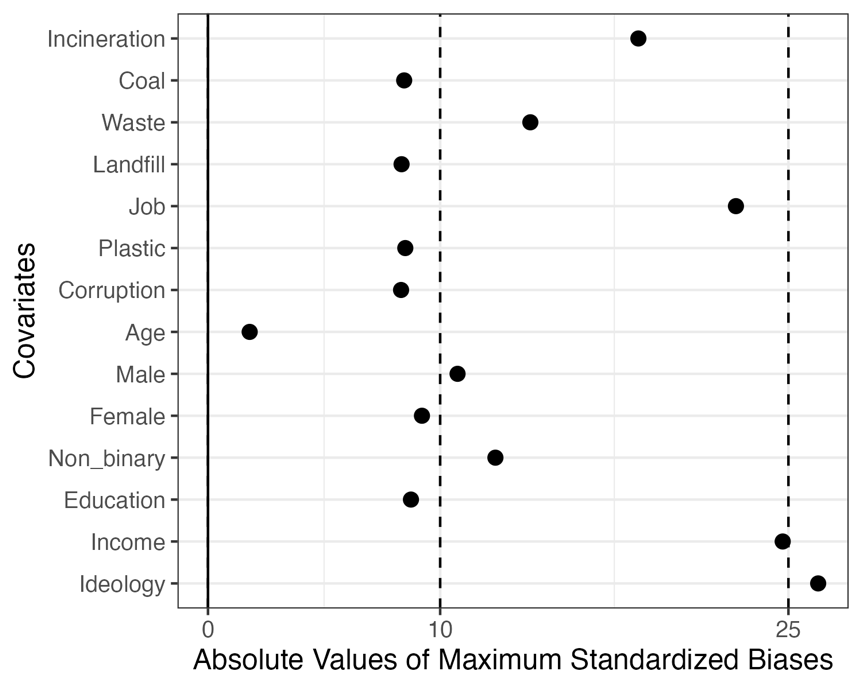


**Figure A2. Geographical distribution of inattentive respondents**

**Inattentive respondents**


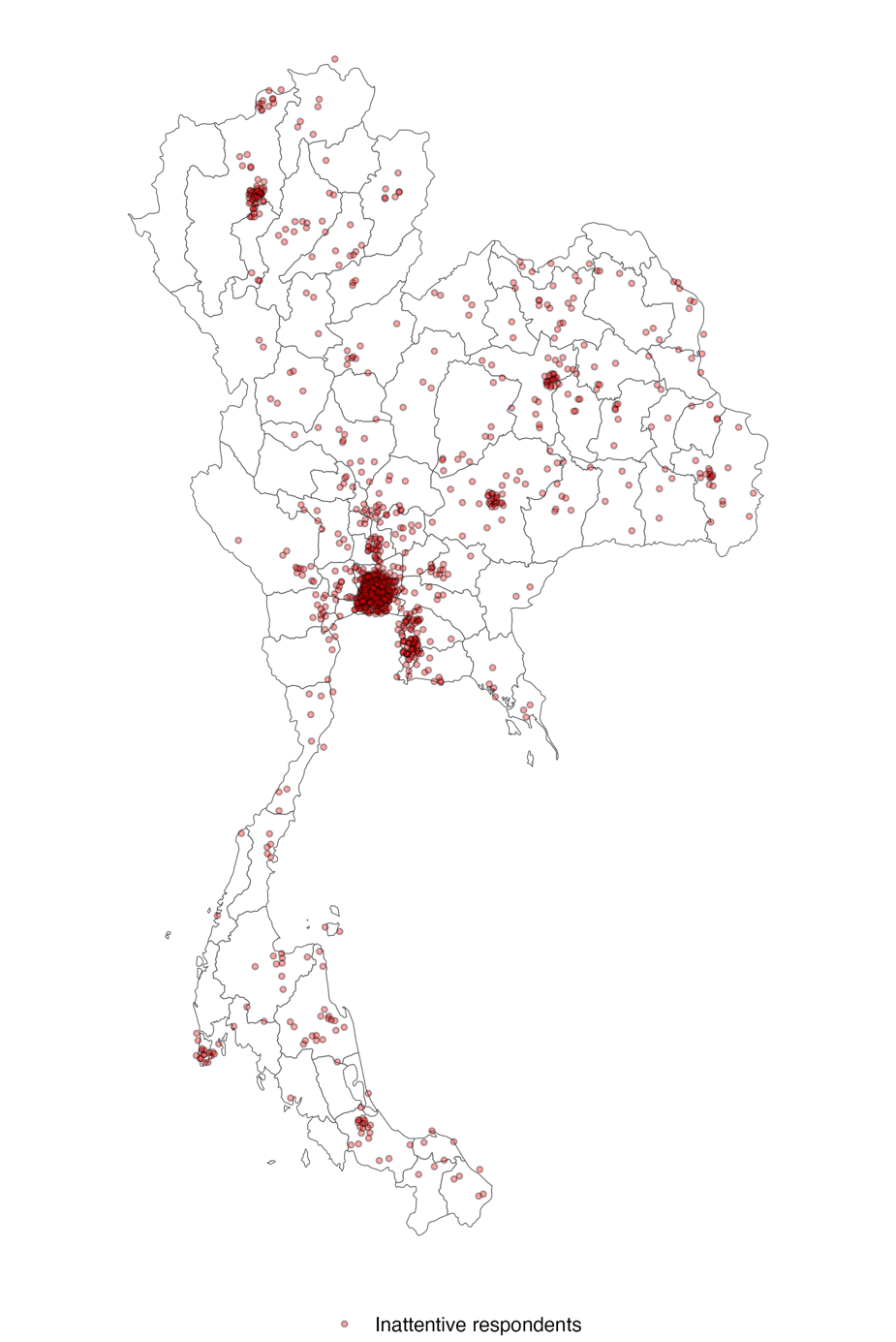


**Figure A3. Geographical location of coal-fired power plants, landfills, and waste incineration facilities**


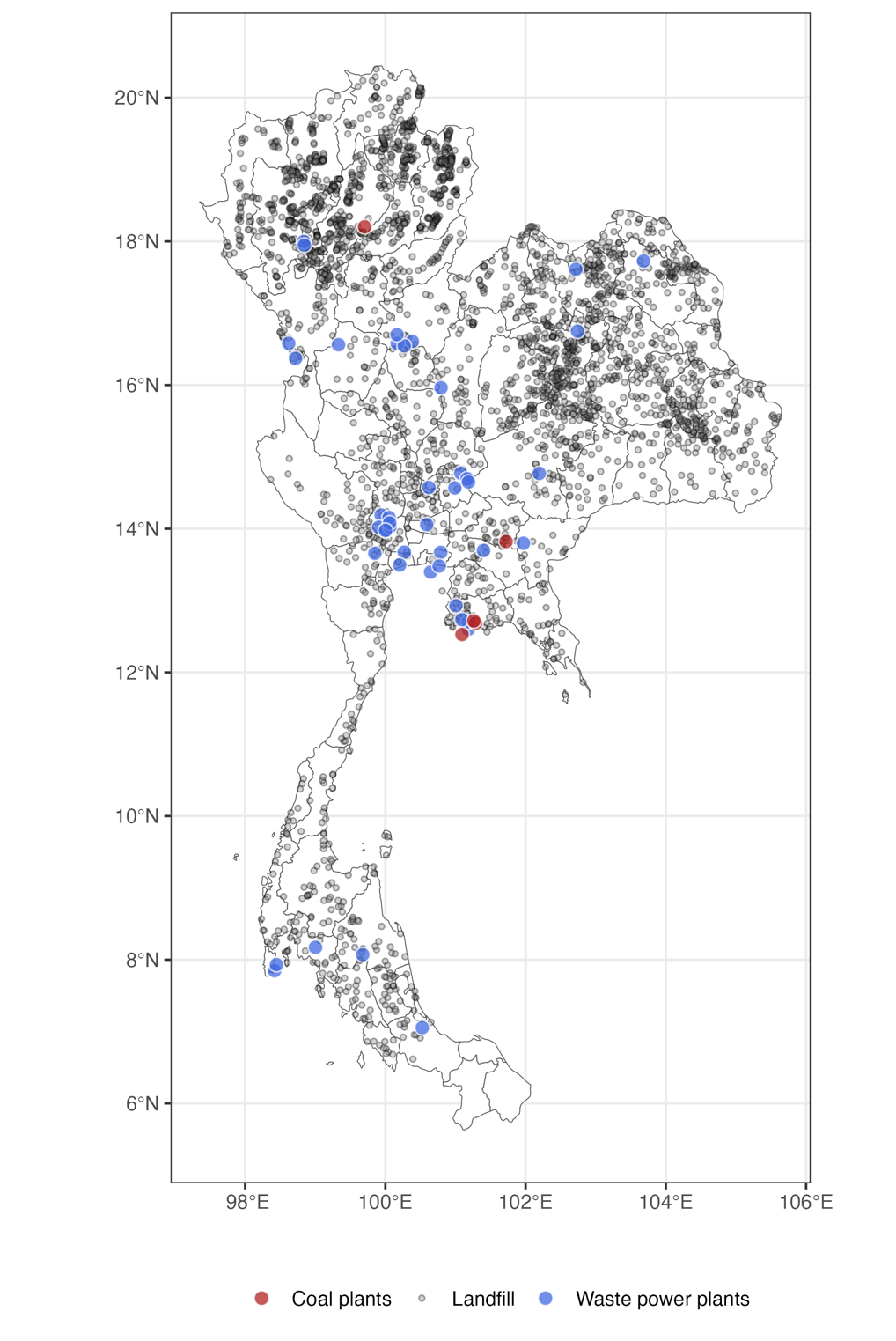


**Table A1. Comparison of demographic distribution between our sample and the census data by age and gender**

| **Age** | **Gender** | **Census (%)** | **Sample (All) (%)** | **Sample (Attentive) (%)** |
| --- | --- | --- | --- | --- |
| 18-29 | Female | 11.7 | 8.8 | 6.8 |
| 18-29 | Male | 11.7 | 15.0 | 14.3 |
| 30-49 | Female | 22.3 | 22.1 | 20.5 |
| 30-49 | Male | 21.3 | 25.1 | 26.2 |
| 50-69 | Female | 13.4 | 14.7 | 17.0 |
| 50-69 | Male | 12.1 | 10.2 | 11.5 |
| 70- | Female | 4.3 | 2.7 | 2.8 |
| 70- | Male | 3.2 | 1.2 | 0.8 |

**Table A2. Results based on the weighted data**

|  |  | | |
| --- | --- | --- | --- |
| (Intercept) | 1.722 | (0.355) | ^***^ |
| Japan | 0.008 | (0.107) |  |
| China | -0.369 | (0.119) | ^**^ |
| Incineration | -0.001 | (0.059) |  |
| Coal | 0.244 | (0.048) | ^***^ |
| Waste | -0.024 | (0.060) |  |
| Landfill | -0.128 | (0.057) | ^*^ |
| Job | 0.581 | (0.056) | ^***^ |
| Plastic | 0.315 | (0.037) | ^***^ |
| Corruption | -0.178 | (0.048) | ^***^ |
| Age | 0.001 | (0.003) |  |
| Gender: male | 0.191 | (0.093) | ^*^ |
| Education | -0.043 | (0.031) |  |
| Income | 0.094 | (0.022) | ^***^ |
| Ideology | -0.105 | (0.035) | ^**^ |
| N | 826 | | |
| AIC | 2841.4 | | |
| RMSE | 1.27 | | |

*Note*: Cluster-robust standard errors in parentheses; * p < 0.05, ** p < 0.01, *** p < 0.001

**Text A1. Full text of survey questions**

**(English translation)**

**Introduction page**

**Welcome to this survey!**

We want to understand your views about the construction of Waste-to-Energy plants in your neighborhood. These plants use plastic waste as a fuel to generate electricity but also create local air pollution. The survey should take around 10-12 minutes to complete. It will be used solely for academic research and is not funded by any commercial or governmental organization.

The survey is anonymous. The information you provide will not be stored or used in any way that could reveal your identity. There are no known risks posed by participating in this survey. The survey has been reviewed and approved by our university’s Human Subject Division. Your participation is voluntary, and you may discontinue participating in the survey at any time.

This survey will produce meaningful results only if you read the questions carefully and express your true opinion.

If you have any questions about this study, please email XXXX. By completing this survey, you are consenting to participate in this study. We are grateful for your participation.

**Age**

How old are you?

If below 18, exit the survey.

**Experimental frames**

Please read the text below **very carefully** since we will ask about the text on the following pages.

Economic growth, urbanization, and population growth have accelerated the production and consumption of single-use plastics and pose a challenge for Thailand’s waste management system. Landfills have been a convenient and cheap way for plastic disposal, but **there is less space to build new landfills to manage the growing volume of plastic waste.**

However, **there is an alternative waste management system as well, known as waste-to-energy (WtE) technology**. By using plastic waste as a fuel for generating electricity, WtE plants eliminate the physical burden of plastic waste while meeting the growing energy demand. Shifting to WtE can reduce various types of pollution caused by landfills, including soil and underground water, which have a detrimental effect on wildlife and humans. Moreover, when landfill capacity is exhausted, plastics get dumped in lakes, rivers, and oceans. **Thus, WtEs can address multiple pollution problems and at the same time, contribute to local economic development by creating new jobs and investment opportunities.**

While some WtE plants already operate in Thailand, the government is encouraging the construction of new ones. **[Insert one of the frames here.]** However, **WtE plants are criticized for contributing to climate change and creating local air pollution. Moreover, burning plastic waste creates toxins which harm human health.** Of course, pollution levels might depend on how air regulations are enforced, what type of technology these plants use, and how well they are managed. Nevertheless, WtE plants need to be carefully evaluated for their benefits and costs.

**Control**: These plants will use locally developed technology in Thailand and will be operated by local Thai companies.

**Frame 1**: These plants will use Japanese technology and will be operated by Japanese companies.

**Frame 2**: These plants will use Chinese technology and will be operated by Chinese companies.

**Main question**

Suppose there is a proposal to construct a new WtE plant using the above-mentioned technology in your local community. Please indicate your support for this project by moving the slider bar.

Strongly oppose Neither support nor oppose Strongly support

1 2 3 4 5 6 7

**Attention and manipulation checks**

The next set of questions is about the text you have just read.

**A1 (for all):**

The construction of what kind of plant or project is under consideration?

Waste-to-energy plant

Waste sorting plant

Waste recycling plant

**A2 (for all):**

Who will operate new plants?

Japanese companies

Chinese companies

Local Thai companies

**Additional questions**

**Plastic pollution**

Do you support the government’s recent ban on the manufacturing and sale of single-use plastics such as plastic bags, plastic utensils, and plastic containers?

Strongly oppose Neither support nor oppose Strongly support

1 2 3 4 5

**Perceived proximity**

To the best of your knowledge, are any waste incineration facilities located in your local community?

- To the best of your knowledge, are any landfills located in your local community?

Yes

No

Don’t know

**Perceptions about local pollution**

How much do you agree/disagree with the following statement?

- My local community experiences air pollution from waste incineration.
- My local community experiences air pollution from coal-fired power plants.
- My local community experiences the plastic waste problem.
- My local community experiences odor, noise, or hygiene issues due to soil and water pollution from landfills.

Strongly agree

Agree

Neither agree nor disagree

Disagree

Strongly disagree

**Perception about corruption**

How much do you agree/disagree with the following statement?

- The national government does not enforce environmental laws.
- My local government does not enforce environmental laws.
- Most local factories in Thailand do not follow environmental laws.

Strongly agree

Agree

Neither agree nor disagree

Disagree

Strongly disagree

**Perception about technologies**

How much do you agree/disagree with the following statement?

- Japanese engineering and technical products such as cars and electronics are of high quality and reliable.
- Chinese engineering and technical products such as cars and electronics are of high quality and reliable.
- Thai engineering and technical products such as cars and electronics are of high quality and reliable.

Strongly agree

Agree

Neither agree nor disagree

Disagree

Strongly disagree

**Perception about pollution levels in home countries**

How much do you agree/disagree with the following statement?

- Japan has strict environmental laws that are strictly enforced.
- China has strict environmental laws that are strictly enforced.
- Thailand has strict environmental laws that are strictly enforced.

Strongly agree

Agree

Neither agree nor disagree

Disagree

Strongly disagree

**Perception about companies’ compliance**

How much do you agree/disagree with the following statement?

- Companies in Japan tend to abide by pollution control laws.
- Companies in China tend to abide by pollution control laws.
- Companies in Thailand tend to abide by pollution control laws.

Strongly agree

Agree

Neither agree nor disagree

Disagree

Strongly disagree

**Perception about economic benefits**

How much do you agree/disagree with the following statement?

WtE plants contribute to local economic development by creating new jobs and investment opportunities.

Strongly agree

Agree

Neither agree nor disagree

Disagree

Strongly disagree

**Gender**

What is your gender?

Male

Female

Non-binary

Prefer not to answer

**Region (region)**

Which district do you currently live in? Please select your region, province, and then district from the pull-down list.

**Postal code**

Which ZIP code do you currently live in? Please put your ZIP code of 5 digits.

**Religion**

What is your religious denomination?

Christianity

Muslim

Buddhism

Other faiths

Not religious

Don’t know/prefer not to answer

**Education**

What is your completed highest level of education?

Primary education or lower

Lower secondary education

Upper secondary education

Vocational certificate

Higher vocational certificate/Certificate of technical vocation/Diploma

Bachelor’s degree

Higher than bachelor’s degree

Don’t know/prefer not to answer

**Occupation**

What is your occupation?

Government employee

State enterprise employee

Private enterprise employee

Self-employed

Merchant/vendor

Service

Agriculture

Laborer

Student

Unemployed

Other

Don’t know/prefer not to answer

**Household income**

Please answer your **household monthly income** (before taxes).

Like the rest of the survey, this question is completely confidential and will be used only to classify the survey responses.

Lower than 5,000 baht

5,001 - 15,000 baht

15,001 - 25,000 baht

25,001 - 35,000 baht

35,001 - 45,000 baht

45,001 - 55,000 บาท 45,001 - 55,000 baht

55,001 - 65,000 baht

65,001 - 75,000 baht

Higher than 75,000 baht

Don’t know/prefer not to answer

**Political ideology**

How much do you agree/disagree with the following statement?

The military should govern the country.

Strongly agree

Agree

Neither agree nor disagree

Disagree

Strongly disagree

**Thank you very much for your cooperation!**

**(Original Thai version)**

วัตถุประสงค์ของการสำรวจ: การสำรวจนี้มีวัตถุประสงค์เพื่อการวิจัยเชิงวิชาการเท่านั้น ไม่มีวัตถุประสงค์เชิงพาณิชย์ เป้าหมายหลักของการสำรวจ คือ เพื่อวัดทัศนคติของผู้มีสิทธิเลือกตั้งเกี่ยวกับประเด็นทางสังคม และเพื่อให้เห็นถึงปัจจัยส่วนบุคคลและปัจจัยด้านสิ่งแวดล้อมที่ส่งผลต่อทัศนคติเหล่านั้น

ความยินยอมในการเข้าร่วม: การเข้าร่วมการสำรวจนี้เป็นไปด้วยความสมัครใจ ท่านสามารถเข้าร่วมการสำรวจนี้ได้เพียงครั้งเดียว หากท่านตกลงเข้าร่วม โปรดคลิก "เริ่มการสำรวจ" ด้านล่าง หากท่านไม่ตกลง โปรดปิดหน้าต่างนี้

การถอนตัว: คำถามบางข้ออาจยากที่จะตอบ หากท่านรู้สึกไม่สะดวกใจ ท่านสามารถเลิกทำแบบสำรวจนี้ได้ทุกเมื่อ หากท่านเลิกทำแบบสำรวจ ท่านจะไม่ได้รับรางวัล แต่ท่านจะไม่ได้รับโทษหรือสูญเสียผลประโยชน์แต่อย่างใด

หมายเหตุ: คำตอบที่ไม่เหมาะสมอาจส่งผลต่อคุณภาพของผลสำรวจ เราขอให้ท่านให้ความร่วมมือตอบคำถามทุกข้อตามความเป็นจริง

เวลาที่คาดว่าจะใช้: แบบสำรวจนี้ใช้เวลาประมาณ 10 นาที

ไม่มีการเสียประโยชน์: แบบสำรวจนี้ไม่เกี่ยวข้องกับการสนับสนุนหรือต่อต้านมาตรการทางการเมืองใดโดยเฉพาะ ท่านจะไม่เสียประโยชน์ใด ๆ จากการเข้าร่วมการสำรวจนี้

การจัดการข้อมูลส่วนบุคคล: คำตอบที่รวบรวมได้จะถูกประมวลผลเพื่อวัตถุประสงค์เชิงสถิติเท่านั้น โปรดมั่นใจว่าจะไม่มีการเปิดเผยตัวตนของผู้ตอบแบบสำรวจ ข้อมูลส่วนบุคคลของท่านจะไม่ถูกเปิดเผยในทางใดทั้งสิ้น

ข้าพเจ้าได้อ่านข้อความนี้แล้วและตกลงเข้าร่วมการสำรวจนี้

**Experimental frames**

โปรดอ่านข้อความด้านล่าง**โดยละเอียด** เนื่องจากจะมีการถามเกี่ยวกับข้อความดังกล่าวในหน้าถัดไป

การเติบโตทางเศรษฐกิจ การขยายตัวของเมือง และจำนวนประชากรที่เพิ่มขึ้นทำให้การผลิตและการบริโภคพลาสติกชนิดใช้ครั้งเดียวเพิ่มขึ้นอย่างรวดเร็ว และเป็นความท้าทายต่อระบบการจัดการขยะของประเทศไทย การฝังกลบเป็นวิธีการที่สะดวกและราคาถูกในการกำจัดขยะพลาสติก **แต่**พื้นที่สำหรับสร้างหลุมฝังกลบเพื่อจัดการขยะพลาสติกที่เพิ่มขึ้นนั้นมีน้อยลง

อย่างไรก็ตาม ยังมีระบบจัดการขยะทางเลือกที่เรียกว่าเทคโนโลยีพลังงานขยะ (waste-to-energy หรือ WtE) ด้วยการใช้ขยะพลาสติกเป็นเชื้อเพลิงในการผลิตกระแสไฟฟ้า โรงไฟฟ้าพลังงานขยะสามารถกำจัดภาระทางกายภาพที่เกิดจากขยะพลาสติก และตอบสนองความต้องการพลังงานที่เพิ่มขึ้น การเปลี่ยนไปใช้เทคโนโลยีพลังงานขยะสามารถลดมลพิษหลากหลายชนิดที่เกิดจากการฝังกลบ ซึ่งรวมถึงมลพิษต่อดินและน้ำใต้ดินซึ่งมีผลกระทบที่เป็นอันตรายต่อสัตว์ป่าและมนุษย์ ยิ่งไปกว่านั้น เมื่อพื้นที่ฝังกลบเต็ม พลาสติกจะถูกทิ้งลงทะเลสาบ แม่น้ำ และมหาสมุทร ด้วยเหตุนี้ เทคโนโลยีพลังงานขยะจึงสามารถแก้ปัญหามลพิษต่าง ๆ และยังมีส่วนช่วยพัฒนาเศรษฐกิจท้องถิ่น ด้วยการสร้างงานและโอกาสในการลงทุน

แม้ว่าจะมีโรงไฟฟ้าพลังงานขยะอยู่บ้างแล้วในประเทศไทย และรัฐบาลก็ยังสนับสนุนให้มีการสร้างเพิ่มขึ้น**[ ]**อย่างไรก็ตาม **โรงไฟฟ้าพลังงานขยะถูกวิพากษ์วิจารณ์ว่าทำให้เกิดการเปลี่ยนแปลงภูมิอากาศและมลพิษทางอากาศในพื้นที่ ยิ่งไปกว่านั้น การเผาขยะพลาสติกยังทำให้เกิดสารพิษซึ่งเป็นอันตรายต่อสุขภาพมนุษย์** แน่นอนว่าระดับมลพิษอาจขึ้นอยู่กับการบังคับใช้กฎหมายเกี่ยวกับคุณภาพอากาศ ชนิดของเทคโนโลยีที่โรงไฟฟ้าใช้ และวิธีการจัดการว่าดีเพียงใด อย่างไรก็ตาม การประเมินประโยชน์และต้นทุนของโรงไฟฟ้าพลังงานขยะอย่างรอบคอบนั้นยังคงจำเป็น

Control: โรงไฟฟ้าเหล่านี้จะใช้เทคโนโลยีที่พัฒนาขึ้นในไทยและจะบริหารจัดการโดยบริษัทไทย

Frame 1: โรงไฟฟ้าเหล่านี้จะใช้เทคโนโลยีญี่ปุ่นและจะบริหารจัดการโดยบริษัทญี่ปุ่น

Frame 2: โรงไฟฟ้าเหล่านี้จะใช้เทคโนโลยีจีนและจะบริหารจัดการโดยบริษัทจีน

**Main question**

สมมติว่ามีข้อเสนอการก่อสร้างโรงไฟฟ้าพลังงานขยะแห่งใหม่โดยใช้เทคโนโลยีที่กล่าวถึงข้างต้นในชุมชนท้องถิ่นของท่าน โปรดระบุว่าท่านจะสนับสนุนโครงการนี้ในระดับใดโดยเลื่อนแถบด้านล่าง

ไม่สนับสนุนอย่างยิ่ง 　　　　 เฉย ๆ 　　　　　　　 สนับสนุนอย่างยิ่ง

1 2 3 4 5 6 7

**Attention and manipulation checks**

ชุดคำถามต่อไปนี้เกี่ยวกับข้อความที่ท่านได้อ่านจบไป

**A1 (for all):**

กำลังมีการพิจารณาก่อสร้างโรงงานหรือโครงการใด

โรงไฟฟ้าพลังงานขยะ

โรงงานคัดแยกขยะ

โรงงานรีไซเคิลขยะ

**A2 (for all):**

ใครจะเป็นผู้บริหารจัดการโรงงานใหม่

บริษัทญี่ปุ่น

บริษัทจีน

บริษัทไทย

**Additional questions**

**Plastic pollution**

ท่านสนับสนุนนโยบายใหม่ของรัฐบาลที่ห้ามผลิตและจำหน่ายพลาสติกชนิดใช้ครั้งเดียว เช่น ถุง อุปกรณ์รับประทานอาหาร และภาชนะพลาสติกหรือไม่

ไม่สนับสนุนอย่างยิ่ง 　　　　 เฉย ๆ 　　　　　　　 สนับสนุนอย่างยิ่ง

1 2 3 4 　 5

**Perceived Proximity**

- เท่าที่ท่านทราบ มีโรงเผาขยะตั้งอยู่ในชุมชนท้องถิ่นของท่านใช่หรือไม่

ใช่

ไม่ใช่

ไม่ทราบ

- เท่าที่ท่านทราบ มีหลุมฝังกลบขยะอยู่ในชุมชนท้องถิ่นของท่านใช่หรือไม่

ใช่

ไม่ใช่

ไม่ทราบ

**Perceptions about local pollution**

ท่านเห็นด้วยหรือไม่เห็นด้วยกับข้อความต่อไปนี้มากน้อยเพียงใด

- ชุมชนท้องถิ่นของฉันประสบปัญหามลพิษทางอากาศจากการเผาขยะ
- ชุมชนท้องถิ่นของฉันประสบปัญหามลพิษทางอากาศจากโรงไฟฟ้าพลังงานถ่านหิน
- ชุมชนท้องถิ่นของฉันประสบปัญหาขยะพลาสติก
- ชุมชนท้องถิ่นของฉันประสบปัญหาเกี่ยวกับกลิ่น เสียง หรือสุขอนามัย เนื่องจากมลพิษทางดินและน้ำจากการฝังกลบขยะ

เห็นด้วยอย่างยิ่ง

เห็นด้วย

เฉย ๆ

ไม่เห็นด้วย

ไม่เห็นด้วยอย่างยิ่ง

**Perception about corruption**

- รัฐบาลกลางไม่ได้บังคับใช้กฎหมายสิ่งแวดล้อม
- รัฐบาลท้องถิ่นของฉันไม่ได้บังคับใช้กฎหมายสิ่งแวดล้อม
- โรงงานในไทยส่วนใหญ่ไม่ได้ปฏิบัติตามกฎหมายสิ่งแวดล้อม

**Perception about technologies**

ท่านเห็นด้วยหรือไม่เห็นด้วยกับข้อความต่อไปนี้มากน้อยเพียงใด

- วิศวกรรมและผลิตภัณฑ์ญี่ปุ่น เช่น รถยนต์ เครื่องใช้ไฟฟ้า มีคุณภาพสูงและน่าเชื่อถือ
- วิศวกรรมและผลิตภัณฑ์จีน เช่น รถยนต์ เครื่องใช้ไฟฟ้า มีคุณภาพสูงและน่าเชื่อถือ
- วิศวกรรมและผลิตภัณฑ์ไทย เช่น รถยนต์ เครื่องใช้ไฟฟ้า มีคุณภาพสูงและน่าเชื่อถือ

เห็นด้วยอย่างยิ่ง

เห็นด้วย

เฉย ๆ

ไม่เห็นด้วย

ไม่เห็นด้วยอย่างยิ่ง

**Perception about pollution levels in home countries**

ท่านเห็นด้วยหรือไม่เห็นด้วยกับข้อความต่อไปนี้มากน้อยเพียงใด

- ญี่ปุ่นมีกฎหมายสิ่งแวดล้อมที่เข้มงวด ซึ่งบังคับใช้อย่างเคร่งครัด
- จีนมีกฎหมายสิ่งแวดล้อมที่เข้มงวด ซึ่งบังคับใช้อย่างเคร่งครัด
- ไทยมีกฎหมายสิ่งแวดล้อมที่เข้มงวด ซึ่งบังคับใช้อย่างเคร่งครัด

เห็นด้วยอย่างยิ่ง

เห็นด้วย

เฉย ๆ

ไม่เห็นด้วย

ไม่เห็นด้วยอย่างยิ่ง

**Perception about companies**

ท่านเห็นด้วยหรือไม่เห็นด้วยกับข้อความต่อไปนี้มากน้อยเพียงใด

- บริษัทในประเทศญี่ปุ่นมีแนวโน้มที่จะปฏิบัติตามกฎหมายควบคุมมลพิษ
- บริษัทในประเทศจีนมีแนวโน้มที่จะปฏิบัติตามกฎหมายควบคุมมลพิษ
- บริษัทในประเทศไทยมีแนวโน้มที่จะปฏิบัติตามกฎหมายควบคุมมลพิษ

เห็นด้วยอย่างยิ่ง

เห็นด้วย

เฉย ๆ

ไม่เห็นด้วย

ไม่เห็นด้วยอย่างยิ่ง

**Perception about economic benefits**

ท่านเห็นด้วยหรือไม่เห็นด้วยกับข้อความต่อไปนี้มากน้อยเพียงใด

**โรงไฟฟ้าพลังงานขยะมีส่วนช่วยพัฒนาเศรษฐกิจท้องถิ่น ด้วยการสร้างงานและโอกาสในการลงทุน**

เห็นด้วยอย่างยิ่ง

เห็นด้วย

เฉย ๆ

ไม่เห็นด้วย

ไม่เห็นด้วยอย่างยิ่ง

**Age**

ท่านมีอายุเท่าใด โปรดกรอกอายุของท่าน

**Gender**

เพศของท่านคือ

ชาย

หญิง

นอนไบนารี

ไม่ต้องการตอบ

**Region**

ปัจจุบันท่านอาศัยอยู่ในเขต/อำเภอใด โปรดเลือกภาค จังหวัด และเขต/อำเภอจากรายการด้านล่าง

**Postal code**

ปัจจุบันท่านอาศัยอยู่ในพื้นที่รหัสไปรษณีย์ใด โปรดกรอกรหัสไปรษณีย์ 5 หลัก

**Religion**

ท่านนับถือศาสนาอะไร

คริสต์

อิสลาม

พุทธ

ความเชื่ออื่น ๆ

ไม่มีศาสนา

ไม่ทราบ/ไม่ต้องการตอบ

(Buddhism should come first, then Christianity and Muslim.)

**Education**

ท่านสำเร็จการศึกษาสูงสุดระดับใด

- ประถมศึกษาหรือต่ำกว่า
- มัธยมศึกษาตอนต้น
- มัธยมศึกษาตอนปลาย
- ปวช.
- ปวส./ปวท./อนุปริญญา
- ปริญญาตรี
- สูงกว่าปริญญาตรี
- ไม่ทราบ/ไม่ต้องการตอบ

**Occupation**

ท่านประกอบอาชีพอะไร

- รับราชการ
- พนักงานรัฐวิสาหกิจ
- พนักงานบริษัท
- ธุรกิจส่วนตัว
- ค้าขาย
- บริการ
- เกษตรกร
- กรรมกร
- นักเรียน/นักศึกษา
- ว่างงาน
- อื่น ๆ
- ไม่ทราบ/ไม่ต้องการตอบ

**Household income**

โปรดบอก**รายได้ต่อเดือนของครัวเรือนของท่าน** (ก่อนหักภาษี)

คำถามนี้เป็นความลับเช่นเดียวกับส่วนอื่นของแบบสำรวจ และจะใช้เพื่อจำแนกประเภทคำตอบเท่านั้น

- ต่ำกว่า 5,000 บาท
- 5,001 - 15,000 บาท
- 15,001 - 25,000 บาท
- 25,001 - 35,000 บาท
- 35,001 - 45,000 บาท
- 45,001 - 55,000 บาท
- 55,001 - 65,000 บาท
- 65,001 - 75,000 บาท
- มากกว่า 75,000 บาท
- ไม่ทราบ/ไม่ต้องการตอบ

**Political orientation**

ท่านเห็นด้วยหรือไม่เห็นด้วยกับข้อความต่อไปนี้มากน้อยเพียงใด

- ทหารควรปกครองประเทศ
- รัฐสภาควรเต็มไปด้วยผู้นำที่มีความสามารถ

เห็นด้วยอย่างยิ่ง

เห็นด้วย

เฉย ๆ

ไม่เห็นด้วย

ไม่เห็นด้วยอย่างยิ่ง

ขอบคุณอย่างยิ่งที่ให้ความร่วมมือในการทำแบบสำรวจ
